# METTL3 Promotes Endothelium-Mesenchymal Transition of Pulmonary Artery Endothelial Cells by Regulating TRPC6/Calcineurin/NFAT Signaling Pathways

**DOI:** 10.1155/2023/8269356

**Published:** 2023-02-21

**Authors:** Chunchu Kong, Fuxiu Zhang, Ruicheng Hu, Lile Wang

**Affiliations:** ^1^Department of Respiratory Medicine, Hunan Provincial People's Hospital (The First Affiliated Hospital of Hunan Normal University), Changsha 410016, China; ^2^Department of Rehabilitative Medicine, Hunan Provincial People's Hospital (The First Affiliated Hospital of Hunan Normal University), Changsha 410016, China

## Abstract

**Background:**

Endothelium-mesenchymal transition (EndMT) is a process of phenotypic and functional transition from activated endothelial cells to mesenchymal cells. Recently, EndMT has been proved to be one of the main pathological mechanisms of pulmonary artery hypertension (PAH). However, the molecular mechanism is not clear.

**Methods:**

Primary rat pulmonary arterial endothelial cells (rPAECs) were isolated from Sprague-Dawley rats and verified by CD31 immunofluorescence staining. rPAECs were exposed to hypoxic conditions to induce EndMT. RNA and protein levels in cells were detected by RT-qPCR and Western blot. The migration ability was verified by the transwell assay. The RIP experiment was used to test the m6A modification of TRPC6 mRNA and the binding relationship between TRPC6 and METTL3. Calcineurin/NFAT signaling was measured by using commercial kits.

**Results:**

METTL3 was found to be highly expressed by hypoxia treatment in a time-dependent manner. Knockdown of METTL3 significantly suppressed cell migration, downregulated the levels of interstitial cell-related markers like *α*-SMA and vimentin, and increased the levels of endothelial cell markers including CD31 and VE-cadherin. Mechanistically, METTL3 increased TRPC6 expression by enhancing the m6A modification of TRPC6 mRNA, thus activating calcineurin/NFAT signaling. Our experiments showed that METTL3 silencing mediated the inhibitory roles in the hypoxia-mediated EndMT process, which were significantly reversed by TRPC6/calcineurin/NFAT signaling activation.

**Conclusion:**

Our results elucidated that METTL3 knockdown inhibited the hypoxia-mediated EndMT process by inactivating TRPC6/calcineurin/NFAT signaling.

## 1. Introduction

Pulmonary artery hypertension (PAH) is a serious and fatal pulmonary vascular disease featured by a continuous increase in pulmonary vascular pressure and pulmonary vasoconstriction and remodeling [1]. PAH is a common cause of the excessive right ventricular pressure load, which can easily develop into right ventricular hypertrophy or right heart failure [2]. Endothelial mesenchymal transition (EndMT) is a complex biological process in which endothelial cells transition to an interstitial phenotype, accompanied by loss of vascular endothelial cell markers including CD31, VE-cadherin, and VEGFR 1/2 and increased interstitial cell markers like *α*-SMA and collagen I/III [3, 4]. Studies have shown that inhibition of EndMT in pulmonary arterial endothelial cells can delay or prevent the occurrence of PAH [5, 6]. Therefore, it is of great significance to explore the molecular mechanism of EndMT and find effective therapeutic targets for PAH.

N6-methyladenosine (m6A) is the most abundant modification type of eukaryotic RNA, which can affect mRNA stability, nuclear export, translation, splicing, and degradation [7]. m6A is a dynamic and reversible process which can be catalyzed by the methyltransferase complex, erased by demethylases, and identified by several RNA-binding proteins [8]. In general, the methyltransferase complex, also called as “m6A writers,” mainly consists of methyltransferase-like 3/14 (METTL3/14), Vir-like m6A methyltransferase-associated (VIRMA) subunits, Wilms tumor 1-associated protein (WTAP), and RNA-binding motif protein 15 (RBM15). Demethylase progress is mediated by FTO and alkB homolog 5 (ALKBH5), which are also known as “m6A erasers.” In addition, m6A recognition is mediated by YTH-domain-containing family proteins (YTHDFs), heterogeneous nuclear ribonucleoproteins (HNRNPs), and insulin-like growth factor 2 mRNA-binding proteins (IGF2BPs), which are all called as “m6A readers.” Emerging pieces of evidence have shown that m6A modification was closely involved in PAH pathology [9–11]. For instance, METTL3 destabilized PTEN mRNA via an m6A modification-dependent manner to accelerate the proliferation and migration of pulmonary artery smooth muscle cells (PASMCs) [9], suggesting that METTL3 was involved in PAH development. Noticeably, METTL3 was also reported to modulate endothelial cell phenotypes. Yao et al. reported that METTL3 was overexpressed in hypoxia-induced HUVECs and accelerated the proliferation, migration, and angiogenesis of HUVECs [12]. In addition, high glucose treatment reduced METTL3 expression, and its overexpression remarkably alleviated high glucose-induced EndMT [13]. Whereas whether METTL3 is related to EndMT in the pathological process of PAH deserves further investigation.

Transient receptor potential cation channel 6 (TRPC6) is a member of the TRPC family which is able to regulate intracellular Ca^2+^ levels. Several studies have confirmed that TRPC6 is involved in the regulation of VMCs and endothelial cell function [14, 15]. The work of Tauseef et al. showed that TRPC6 activation could enhance endothelial cell permeability by inducing Ca^2+^ influx, destroy vascular endothelial barriers, and promote pulmonary vascular injury [16]. Jain et al. demonstrated that TRPC6 was upregulated in PAMSCs isolated from PAH patients, and inhibition of TRPC6 by BI-749327 could inactivate PI3K/AKT/mTOR pathways to restore pulmonary vascular remodeling [17]. Calcineurin is a Ca^2+^/CAM-dependent serine and threonine phosphorylase, which can dephosphorylate nuclear factor of activated T (NFAT) family transcription factors in the cytosol and then transfer to the cell nucleus to regulate the transcription of related genes [18]. Previous reports have uncovered that calcineurin/NFATs signaling aggravated the migration and proliferation of PAMSCs and pulmonary artery remodeling to promote PAH progression [19, 20]. It has been confirmed that TRPC6-mediated Ca^2+^ influx could sustain activated calcineurin/NFAT signaling pathways [21]. Whereas the detailed regulatory mechanism of TRPC6/calcineurin/NFATs pathways in PAH remains unexplored.

Here, we aimed to investigate the roles and mechanism of METTL3-mediated m6A modification in hypoxia-induced EndMT. Intriguingly, we observed that a highly probable m6A modification site (-GGACU-) was found in TRPC6 mRNA sequence through SRAMP online bioinformatic tools. Therefore, it is reasonable to speculate that METTL3-mediated m6A modification might regulate hypoxia-induced EndMT via TRPC6/calcineurin/NFAT pathways.

## 2. Materials and Methods

### 2.1. Cell Isolation, Culture, and Treatment

Male Sprague-Dawley (180–220 g) rats provided by the Laboratory Animal Centre of Guangdong (Guangzhou, China) were used to isolate pulmonary arterial endothelial cells (rPAECs) as previously described [22]. All experimental procedures were approved by Hunan Provincial People's Hospital. The isolated rPAECs were grown in Dulbecco's modified Eagle's medium (DEME, Gibco, Carlsbad, CA, USA) containing 10% fetal bovine serum (FBS, Gibco), 50 *μ*g/ml streptomycin, and 50 U/ml penicillin. For cell treatment, the rPAECs (3–6 passages) were exposed to 10 *μ*M hyperforin (HF, TRPC6 agonist, ChemeGen, USA) or 10 nM ET-1 (Ca^2+^ activator, Sigma, St. Louis, MO, USA) for 24 h. For hypoxia (Hx) treatment, the rPAECs were transferred to 1% O_2_ for 6, 12, 24, and 48 h when they reached 80% confluency.

### 2.2. Immunofluorescence

The isolated rPAECs were collected and digested by 0.8% trypsin to prepare cell suspension. After centrifugation, the rPAECs were harvested and resuspended by using Hanks' solution. Then, cell suspension was coated on glass slides and further fixed with 4% formaldehyde for 30 min. After permeation with 0.2% Triton X-100 and blocking by 5% BSA, the primary antibody against CD31 (PA5-32321, Invitrogen, Carlsbad, CA, USA) was incubated overnight at 4°C. On the next day, the cells were further incubated with the secondary antibody (A-11006, Invitrogen) for 1 h. DAPI (Beyotime, Shanghai, China) stained cell nuclei for 10 min. Finally, the cells were photographed using a fluorescence microscope (Leica microsystems, IL, USA).

### 2.3. Migration Assay

The migration ability of rPAECs was determined by using a transwell chamber (8 *μ*m, Corning, NY, USA). Briefly, 5 × 10^4^ rPAECs were precultured in a serum medium for 12 h and then resuspended in 200 *μ*L of a serum-free medium and seeded into the upper chamber. Then, 600 *μ*L of the medium containing completely FBS was added into the bottom chamber. After 48 h, the cells in the upper chamber were removed and the migratory cells in the bottom were fixed with 4% paraformaldehyde and stained with crystal violet. Finally, the migratory cells were visualized under a microscope.

### 2.4. Lentivirus Construction and Transfection

Short hairpin RNA (shRNA) targeting METTL3 and shNC were synthesized and inserted into lentivirus vectors (GeneChem, Shanghai, China). Next, recombinant shMETTL3 or shNC-lentivirus vectors and their packaging vectors (pHelper 1.0, GeneChem) were cotransfected into 293T cells by utilizing Lipofectamine 2000 (Invitrogen) to generate viral particles (lv-shMETTL3, lv-shNC). For rPAEC transfection, the rPAECs were placed into a 6-well plate for further incubation. When the cells reached 40% confluence, the cultured medium was replaced with a serum-free medium and the rPAECs were infected with lv-shMETTL3 or lv-shNC vectors at an MOI of 20. After transfection for 12 h, the rPAECs were harvested to detect the transfection efficiency by real-time qPCR (RT-qPCR).

### 2.5. RNA Extraction and the RT-qPCR Assay

The total RNA samples in rPAECs were extracted using TRIzol regents (Invitrogen). After DNase I treatment, cDNA was synthesized by using the PrimeScript reverse transcription kit (TaKaRa, Shiga, Japan). Next, SYBR Premix Ex Taq™ (TaKaRa) was applied for RT-qPCR analysis. The relative mRNA levels of METTL3 and TRPC6 were normalized to those of GAPDH by using the 2^−ΔΔCT^ method. The experimental primers were provided as follows (5′-3′): METTL3 (forward): -CCGCGCTAGGAACTAGGATG, METTL3 (reverse): CCACTAGAGGTAGGGGCAGT; TRPC6 (forward): CAGCCGTTTAAAACTCGCTATT, TRPC6 (reverse): ACCACGAGGAATTTCACTGC; and GAPDH (forward): GCAAGTTCAACGGCACAG, GAPDH (reverse): GCCAGTAGACTCCACGACAT.

### 2.6. Western Blot Assay

Total proteins were extracted from rPAECs with different treatments by using RIPA regents (Beyotime). After quantification by the BCA kit (Beyotime), the equal amounts of proteins were separated by 10% SDS-PAGE and subsequently moved onto the PVDF membrane (Millipore, USA). Next, the membrane was blocked utilizing nonfat milk (5%) for 1 h. The primary antibodies against METTL3 (ab195352, Abcam, Cambridge, MA, USA), TRPC6 (PA5-95049, Invitrogen), CD31 (PA5-32321, Invitrogen), VE-cadherin (ab231227, Abcam), *α*-SMA (14-9760-82, Invitrogen), vimentin (ab92547, Abcam), NFATc1 (MA5-32686, Invitrogen), NFATc2 (MA5-32661, Invitrogen), NFATc3 (PA5-79734, Invitrogen), NFATc4 (PA5-105650, Invitrogen), and GAPDH (ab8245, Abcam) were incubated with the membrane at 4°C. On the next day, the secondary antibody (ab150165, Abcam) was added to the membrane for another 2 h. Afterwards, the protein bands were developed by using the ECL kit (Thermo Fisher Scientific, Waltham, MA, USA). Finally, the intensity of the bands was measured using ImageJ software.

### 2.7. RNA Immunoprecipitation (RIP) Assay

The direct binding relationship between METTL3 and TRPC6 was assessed using a commercial RIP kit (Millipore). In detail, rRAECs (5 × 10^7^) were collected after different treatments and lysed utilizing RIP lysis buffer. The magnetic beads were preincubated with the specific antibodies targeting METTL3 (ab195352, Abcam), m6A (ab286164, Abcam), and mouse IgG (ab207997, Abcam) for overnight. Then, the antibody-bead complex was incubated with the prepared cell lysates at 4°C for 24 h. After proteinase K treatment, precipitated RNA was extracted and subjected to the RT-qPCR assay.

### 2.8. Calcineurin Activity Determination

To measure the changes of calcineurin activity in rPAECs after indicated treatment, Calcineurin Phosphatase Activity Assay Kit (ab139461, Abcam) was used according to the instruction's protocol. Briefly, the cell lysates were mixed with calcineurin assay buffer, followed by addition of calcineurin substrates for incubation for 10 min. Then, the green assay reagent was added into the mixture, and the OD value at 620 nm was recorded by using a microplate reader (BioTek, USK). Calcineurin activity in each group was calculated relative to the normoxia (Nx) group.

### 2.9. Transcriptional Activity of NFAT

To assay the signal transduction of Ca^2+^/calcineurin/NFAT signaling, the commercial recombinant NFAT-Luc plasmids (Yesen, Shanghai, China) were purchased and transfected into rPAECs by using Lipofectamine 2000 (Invitrogen). Then, the transfected rPAECs were divided into six groups for indicated treatment: Nx, Hx, Hx + lv-shNC, Hx + lv-shMETTL3, Hx + HF, and Hx + lv-shMETTL3+HF. After 24 h, the cells were harvested to determine the luciferase activity using a dual luciferase reporter assay system (Promega, Madison, WI, USA).

### 2.10. Data Analysis

All experiments were conducted independently no less than three times. The data were calculated by GraphPad Prism 5.0 and presented as a mean ± standard deviation (SD). All the data were in accordance with normal distribution and homogeneity of the variance test. Unpaired Student's *t*-test was used to compare the data between two groups, and the one-way analysis of variance (ANOVA) followed by Tukey's post hoc was adopted for data comparison of multiple groups. Data were considered statistically significant when *P* value was less than 0.05.

## 3. Results

### 3.1. METTL3 Was Gradually Upregulated in Hypoxia-Induced rPAECs

**Figure 1:**
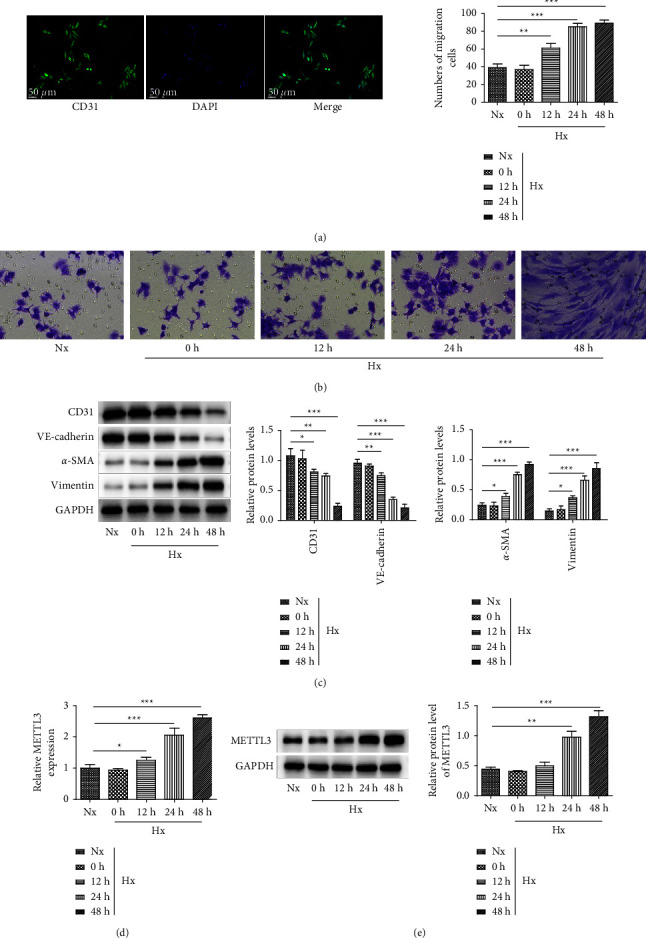
METTL3 was gradually upregulated in hypoxia-induced rPAECs. (a) The primary rPAECs isolated from male Sprague-Dawley rats were identified using immunofluorescence. rPAECs were exposed to hypoxic conditions (Hx, 1% O_2_) for 6 h, 12 h, 24 h, and 48 h. (b) The migration of rPAECs was determined by the transwell assay. (c) Western blot examined the protein levels of CD31, VE-cadherin, *α*-SMA, and vimentin. (d, e) The mRNA and protein levels of METTL3 were determined by RT-qPCR and western blot. ^∗^*P* < 0.05, ^∗∗^*P* < 0.01, and ^∗∗∗^*P* < 0.001.

First, the primary rPAECs were isolated from male Sprague-Dawley rats and identified using a specific antibody against CD31 via an immunofluorescence analysis. As shown in Figure 1(a), the positive rate of the isolated rPAECs was higher than 90%, indicating that the cells were successfully isolated. After hypoxia treatment, we observed that the migration of the rPAECs was increased in a time-dependent manner (Figure 1(b)). Next, the western blot results showed that endothelial cell markers including CD31 and VE-cadherin were downregulated, and the markers of interstitial cells like *α*-SMA and vimentin were increased gradually after hypoxia, which indicated that EndMT was developed after hypoxia treatment (Figure 1(c)). In addition, the levels of METTL3 were found to be enhanced by hypoxia treatment in a time-dependent manner (Figures 1(d) and 1(e)). These findings suggested that METTL3 was upregulated during hypoxia-induced EndMT in rPAECs.

### 3.2. Downregulation of METTL3 Suppressed the Hypoxia-Induced EndMT Process

**Figure 2:**
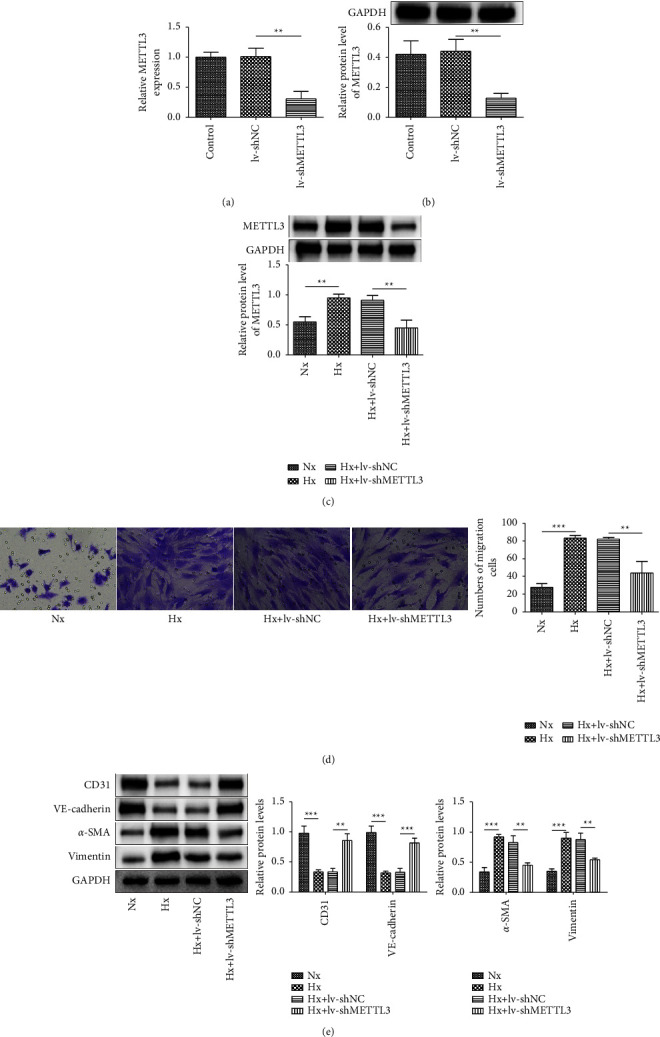
Downregulation of METTL3 suppressed the hypoxia-induced EndMT process. (a, b) Lentivirus-mediated shMETTL3 and shNC were transfected into rPAECs; then, the mRNA and protein levels of METTL3 were determined by RT-qPCR and western blot. lv-shMETTL3- and lv-shNC-transfected cells were exposed to hypoxic conditions. (c) Western blot detected the METTL3 protein level. (d) The migration of rPAECs was evaluated by the transwell assay. (e) Western blot examined the levels of CD31, VE-cadherin, *α*-SMA, and vimentin. ^∗^*P* < 0.05, ^∗∗^*P* < 0.01, and ^∗∗∗^*P* < 0.001.

Lentivirus-mediated shMETTL3 plasmids were transfected into rPAECs to knock down METTL3 expression. As confirmed in Figures 2(a) and 2(b), METTL3 mRNA and protein levels were remarkably reduced upon lv-shMETTL3 plasmid transfection. After hypoxia exposure, the lv-shMETTL3 plasmids greatly reversed the increase of METTL3 in hypoxia-induced rPAECs (Figure 2(c)). Next, hypoxia treatment greatly enhanced the migration of rPAECs, while this effect was significantly reduced by METTL3 knockdown (Figure 2(d)). Compared to the Nx group, the expressions of CD31 and VE-cadherin were suppressed and *α*-SMA and vimentin were enhanced after hypoxia treatment, whereas these trends were greatly abolished by METTL3 knockdown (Figure 2(e)). Overall, these results demonstrated that METTL3 silencing suppressed hypoxia-triggered EndMT.

### 3.3. METTL3 Upregulated TRPC6 Expression in an m6A-Dependent Manner

**Figure 3:**
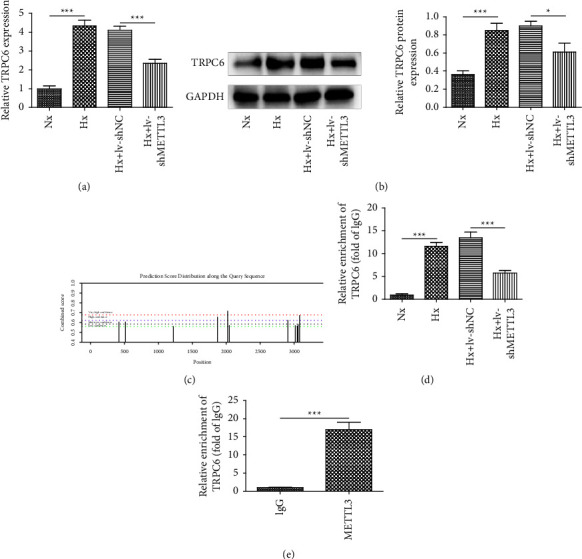
METTL3 upregulated TRPC6 expression in an m6A-dependent manner. lv-shMETTL3- and lv-shNC-transfected cells were exposed to hypoxic condition. (a, b) The mRNA and protein level of TRPC6 were examined using RT-qPCR and western blot. (c) The SRAMP database (http://www.cuilab.cn/sramp) predicted the m6A modification sites of TRPC6 mRNA. (d) The m6A modification level of TRPC6 mRNA was assessed by the meRIP assay. (e) The direct binding relationship between TRPC6 and METTL3 was verified by the RIP assay. ^∗^*P* < 0.05, ^∗∗^*P* < 0.01, and ^∗∗∗^*P* < 0.001.

Recently, a large body of references supported that TRPC6 was a promising therapeutic target for PAH [17, 23]. In this part, we attempted to examine whether METTL3 had an association with TRPC6. As shown in Figures 3(a) and 3(b), TRPC6 mRNA was greatly upregulated in hypoxia-induced rPAECs; notably, these changes were markedly reversed by METTL3 knockdown. Subsequently, the SRAMP database predicted several potential m6A modification sites in the TRPC6 sequence (Figure 3(c)). Intriguingly, compared to the Nx group, the m6A modification level of TRPC6 was enhanced after hypoxia treatment and knockdown of METTL3 significantly downregulated m6A levels of TRPC6 mRNA (Figure 3(d)). Moreover, the abundance of TRPC6 mRNA was determined in the RNA complex pulled down by the specific METTL3 antibody (Figure 3(e)). Thus, these data inferred that METTL3 promoted TRPC6 expression by enhancing m6A modification of TRPC6 mRNA.

### 3.4. METTL3 Activated Calcineurin/NFAT Signaling via Promoting TRPC6

**Figure 4:**
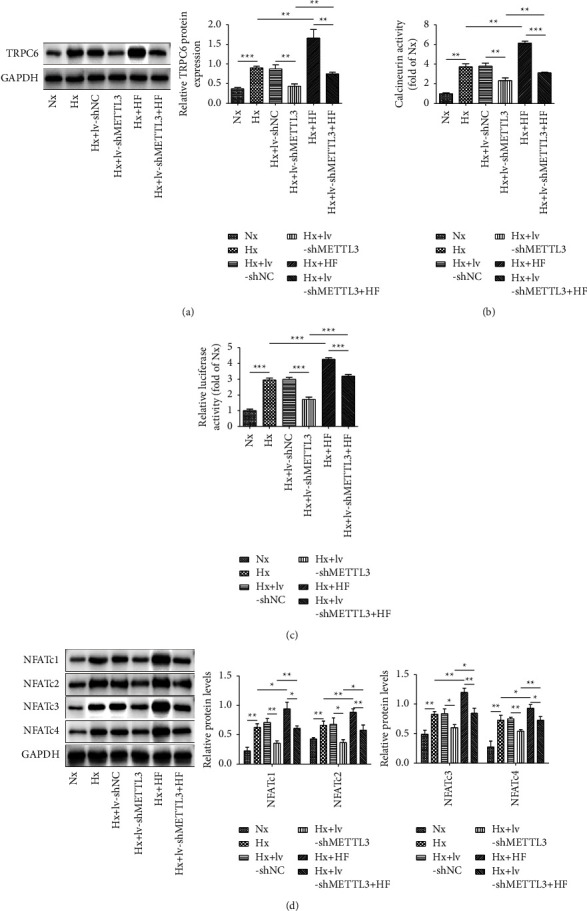
METTL3 activated calcineurin/NFAT signaling via promoting TRPC6. rPAECs were divided into the following groups: Nx, Hx, Hx + lv-shNC, Hx + lv-shMETTL3, Hx + HF, and Hx + lv-shMETTL3 + HF. After indicated treatment, the subsequent experiments were conducted. (a) Western blot assessed the protein level of TRPC6. (b) The changes in calcineurin activity were detected by using the commercial kit. (c) The transcriptional activity of NFAT was detected by the luciferase assay. (d) Western blot quantified the levels of NFATc1, NFATc2, NFATc3, and NFATc4. ^∗^*P* < 0.05, ^∗∗^*P* < 0.01, and ^∗∗∗^*P* < 0.001.

As shown in Figure 4(a), silencing of METTL3 repressed the protein level of TRPC6 in hypoxia-induced rPAECs, while HF treatment greatly enhanced TRPC6 expression, which remarkably reversed the inhibitory role mediated by METTL3 downregulation. Moreover, the calcineurin activity and the NFAT transcription activity were increased in hypoxia-induced rPAECs, and these trends diminished when METTL3 was silenced. Meanwhile, HF treatment resulted in a significant increase in the calcineurin activity and NFAT transcription activity in hypoxia-treated rPAECs, which greatly reversed METTL3 silence-mediated repressive effects (Figures 4(b) and 4(c)). Similarly, the protein levels of NFATc1, NFATc2, NFATc3, and NFATc4 were increased after hypoxia exposure, and METTL3 knockdown markedly reversed the promoting role mediated by hypoxia treatment in these proteins. In contrast, HF treatment further strengthened the promotion of these proteins by hypoxia treatment. Notably, HF treatment strikingly reversed the repressive effect of METTL3 silencing on NFATc1∼4 (Figure 4(d)). Therefore, METTL3 could activate calcineurin/NFAT signaling through enhancing TRPC6 expression.

### 3.5. Activation of TRPC6/Calcineurin/NFAT Pathways on the Biological Roles of METTL3 Knockdown in Hypoxia-Induced rPAECs

**Figure 5:**
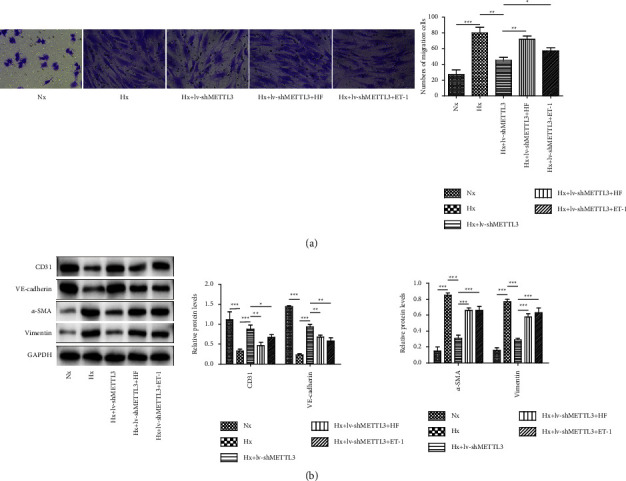
Activation of TRPC6/calcineurin/NFAT pathways on the biological roles of METTL3 knockdown in hypoxia-induced rPAECs. rPAECs were divided into the following groups: Nx, Hx, Hx + lv-shMETTL3, Hx + lv-shMETTL3+HF, and Hx + lv-shMETTL3 + ET-1. After indicated treatment, the subsequent experiments were conducted. (a) The migration of rPAECs was tested by the transwell assay. (b) Western blot examined the levels of CD31, VE-cadherin, *α*-SMA, and vimentin. ^∗^*P* < 0.05, ^∗∗^*P* < 0.01, and ^∗∗∗^*P* < 0.001.

In this section, we aim to investigate the functional association between METTL3 and TRPC6/calcineurin/NFAT signaling. Compared to the hypoxia group, the downregulation of METTL3 obviously reduced the migration of rPAECs, while this effect could be reversed by HF or ET-1 treatment (Figure 5(a)). In addition, the western blot assay presented that METTL3 downregulation mediated the promoting roles in CD31 and VE-cadherin and that the inhibitory roles in vimentin and *α*-SMA were strikingly reversed by HF or ET-1 treatment (Figure 5(b)). These results revealed that the protective roles of METTL3 downregulation in hypoxia-induced EndMT were partially dependent on the inactivation of TRPC6/calcineurin/NFAT pathways.

## 4. Discussion

For several decades, clinical existing drugs have been approved for the treatment of PAH, such as endothelin receptor antagonists, phosphodiesterase inhibitors, and top ring element approaches. [24]. However, these drugs can only slightly balance vascular contraction and expansion, reduce vascular resistance, improve the patients' symptoms, and cannot thoroughly cure PAH. Therefore, there is an urgent need for in-depth understanding of the PAH pathogenesis to develop a new and effective therapeutic drug against PAH. Emerging reports have supported that PAECs undergoing EndMT play a critical role in PAH pathology. Previous reports have shown that hypoxia treatment can enhance the proliferation and migration ability of PAECs while losing endothelial characteristics and changing to mesenchymal phenotypes and functional response, leading to endothelial dysfunction [25–28]. Similar to previous results, we have confirmed that hypoxic induction resulted in a stronger migration ability of rPAECs. Moreover, during hypoxia induction, we found that rPAECs gradually lost endothelial cell-specific surface markers such as CD31 and VE-cadherin while acquiring interstitial cell surface markers such as vimentin and *α*-SMA, suggesting that EndMT was induced by hypoxia. Divertingly, METTL3 was confirmed to be upregulated during EndMT, suggesting that METTL3 might be a key target in the EndMT process of rPAECs.

METTL3 is a core catalytic subunit of the methyltransferase complex which can recognize conserved RNA sequence 5′-RRACU-3′(*R* = *A* or *G*) and catalyzes the methylation of the sixth nitrogen atom of adenine [29, 30]. A large number of pieces of evidence have shown that METTL3 plays a critical regulatory role at the posttranscriptional level, involving in splicing of precursor mRNA, export of mature mRNA, regulation of mRNA stability, etc., thus affecting a variety of biological processes [31, 32]. The Lin et al. report showed that METTL3 was increased in VMC differentiation induced by hypoxia and that downregulation of METTL3 led to the decrease of *α*-SMA, SM22*α*, calponin, and SM-MHC in VMSCs [33]. Qin et al. also revealed that METTL3 was abnormally overexpressed in hypoxia-induced PAH in vivo and vitro and that METTL3 inhibition could suppress the proliferation and migration of PAMSCs through destabilizing PTEN mRNA [9]. These findings suggested that METTL3 could promote PAH occurrence by affecting the excessive proliferation and migration of PAMSCs. In addition, METTL3 is also a key regulator of endothelial cell phenotypic changes. Yao et al. found that METTL3-mediated m6A modifications in mouse retinal endothelial cells increased after hypoxia treatment and that silencing METTL3 significantly reduced endothelial cell proliferation, migration, and angiogenesis [12]. Similarly, hypoxic stimulation could upregulate METTL3-mediated m6A modification in endothelial progenitor cells (EPCs), which could increase the proliferation, migration, and angiogenic activity of EPCs by activating VEGF/PI3K/ AKT pathways [34]. Notably, PI3K/AKT pathways and VEGF protein were confirmed to be closely related to the EndMT process of ECs [35–37], which implied that METTL3 might be involved in the regulation of the EndMT process in rPAECs. Likewise, Cao et al. revealed that METTL3 suppressed the EndMT of high glucose-induced HRMECs to alleviate diabetic retinopathy [13]. They suggested that METTL3 might have different regulatory effects on endothelial cell phenotypes under different conditions. As conjectured above, METTL3 was increased in a time-dependent manner after hypoxia induction. Moreover, hypoxia-induced migration and EndMT of rPAECs were significantly attenuated after METTL3 downregulation, suggesting that METTL3 was a key regulatory molecule in the regulation of PAH pathology.

TRPC family proteins (TRPC1∼7) are nonspecific cation channels of Ca^2+^ in cell membranes, which are involved in the regulation of the smooth muscle and endothelial cell function by binding to G protein-coupled receptors and regulating intracellular Ca^2+^ levels [14, 38]. Previous studies have shown that TRPC6 was upregulated in a chronic hypoxia-induced PAH model and was involved in the regulation of receptor-operated calcium channels (ROCCs) triggering PAMSC proliferation and vasoconstriction [17, 39, 40]. Inhibition of TRPC6 inhibited hypoxia-induced pulmonary vasoconstriction and proliferation and migration phenotypes of PAMSCs, which is a novel therapeutic approach to PAH [41, 42]. The Lang et al. report confirmed that inhibition of TRPC6 could prevent proliferation, migration, and lumen formation of retinal capillary endothelial cells induced by high glucose [43]. However, it is unclear whether TRPC6 regulated EndMT. Here, we first highlighted that TRPC6 was upregulated in hypoxia-induced rPAECs, while TRPC6 could be suppressed upon METTL3 was silenced. After bioinformatic prediction and RIP validation, we confirmed that the m6A modification of TRPC6 mRNA was enhanced in rPAECs after hypoxic induction, which was reversed by METTL3 knockdown. The direct interaction between TRPC6 mRNA and METTL3 protein was subsequently verified. Functional rescue experiments revealed that the suppressive roles of METTL3 downregulation in the migration and EndMT process of hypoxia-induced rPAECs were abolished by TRPC6 agonists, which indicated that TRPC6 positively involved in the regulation of METTL3 in the hypoxia-induced EndMT process.

Calcineurin is a calcium ion-dependent serine and threonine phosphorylase that is activated by increased intracellular calcium concentration and plays a key role in mediating intracellular information transmission [44]. Calcineurin activation can dephosphorylate NFATc1-c4 in the cytosol and promote its translocation into the nucleus to regulate the transcription of related genes [45]. Activation of calcineurin/NFAT signaling pathways has been validated to involve in the pathological process of PAH. For instance, calcineurin/NFAT signaling-mediated cyclin A expression and CDK2 activation contributed to PAMSC proliferation [19]. It has been reported that the calcium-sensing receptor (CaSR) was involved in the high glucose-induced EndMT process [46]. Constitutively active calcineurin A could restore the impaired EndMT process in Tmem100 null AVC explants [47]. Likewise, the detailed roles of calcineurin/NFATs in hypoxia-triggered EndMT remain unclear. It has been reported that TRPC6 and calcineurin/NFAT signaling formed a positive feedback regulatory association [20, 21]. Importantly, our experimental results demonstrated that the calcineurin/NFAT pathway was activated in hypoxia-induced rPAECs, and this trend was significantly reversed and enhanced after METTL3 silencing and TRPC6 activation, respectively. In the rescue experiments, ET-1, an agonist of calcineurin, significantly enhanced the migration and EndMT process of rPAECs, which significantly attenuated the inhibitory effects mediated by METTL3 silencing.

In summary, our study is the first to investigate the function and molecular mechanism of METTL3 in the hypoxia-induced EndMT of rPAECs. The experimental results elucidated that knockdown of METTL3 alleviated hypoxia-induced migration and the EndMT process of rPAECs by inactivation of TRPC6/calcineurin/NFAT signaling. These findings are helpful to provide new insights and intervention targets for the treatment of clinical PAH.

## Data Availability

All data supporting this work are included within the paper on request.

## Abbreviations

EndMT: Endothelium-mesenchymal transition
rPAECs: Rat pulmonary arterial endothelial cells
PAH: Pulmonary artery hypertension
m6A: N6-methyladenosine
METTL3: Methyltransferase-like 3
WTAP: Wilms tumor 1-associated protein
VIRMA: Vir-like m6A methyltransferase-associated subunits
RBM15: RNA-binding motif protein 15
YTHDFs: YTH-domain-containing family proteins
IGF2BPs: Insulin-like growth factor 2 mRNA-binding proteins
HNRNPs: Heterogeneous nuclear ribonucleoproteins
PASMCs: Pulmonary artery smooth muscle cells
TRPC6: Transient receptor potential cation channel 6
NFAT: Nuclear factor of activated T
DEME: Dulbecco's modified Eagle's medium
FBS: Fetal bovine serum
HF: Hyperforin
shRNA: Short hairpin RNA
RT-qPCR: Real-time qPCR
Nx: Normoxia
Hx: Hypoxia
CaSR: Calcium-sensing receptor.

## Additional Points

(1) METTL3 was gradually upregulated in hypoxia-induced rPAECs. (2) Downregulation of METTL3 suppressed the hypoxia-induced EndMT process. (3) METTL3 activated TRPC6/calcineurin/NFAT signaling in an m6A-dependent manner. (4) TRPC6/calcineurin/NFAT signaling activation reversed METTL3 silence-mediated roles in hypoxia-induced rPAECs.
